# Leukemia inhibitory factor promotes EMT through STAT3-dependent miR-21 induction

**DOI:** 10.18632/oncotarget.6756

**Published:** 2015-12-24

**Authors:** Xuetian Yue, Yuhan Zhao, Cen Zhang, Jun Li, Zhen Liu, Juan Liu, Wenwei Hu

**Affiliations:** ^1^ Department of Radiation Oncology, Rutgers Cancer Institute of New Jersey, Rutgers State University of New Jersey, New Brunswick, NJ, USA; ^2^ Department of Pharmacology, Rutgers State University of New Jersey, New Brunswick, NJ, USA

**Keywords:** LIF, miR-21, epithelial-mesenchymal transition, STAT3

## Abstract

Leukemia inhibitory factor (LIF) is a multi-function cytokine. Its role in cancer is not well-understood. Recent studies including ours show that LIF is frequently overexpressed in many types of human tumors and promotes the progression and metastasis of tumors. However, the underlying mechanism of LIF's promoting effects on tumor progression and metastasis is poorly defined. Epithelial-mesenchymal transition (EMT) plays an important role in tumor metastasis. This study reports that LIF promotes EMT in human tumor cells. Overexpression of LIF promotes tumor cells to acquire mesenchymal features, including morphological changes of cells from epithelial-like to mesenchymal-like, increased expression levels of mesenchymal markers and decreased expression of epithelial markers. Knockdown of endogenous LIF reverses EMT in cancer cells. We further identified that LIF induces the expression of microRNA-21 (miR-21), which in turn mediates the promoting effect of LIF on EMT. LIF induces miR-21 expression through the activation of STAT3. Importantly, blocking miR-21 function greatly abolished the promoting effect of LIF on EMT and the migration ability of cancer cells. Taken together, results from this study identified an important function and a novel underlying mechanism of LIF in EMT and tumor metastasis.

## INTRODUCTION

Leukemia inhibitory factor (LIF) is a multi-function cytokine. LIF functions through an autocrine or paracrine manner to bind to its receptor complex, LIF receptor (LIF-R)/gp130, and leads to the activation of many signaling pathways. In different types of tissues and cells, and different stages of development, LIF activates distinct signaling pathways, including JAK/STAT3, MAPK, ERK, AKT and mTOR signaling, etc., and is involved in many important biological functions in neuronal, endocrine, reproductive, inflammatory and immune systems [1–4]. Recent studies including ours have shown that LIF is an important player for the progression and metastasis of different types of solid tumors [3–6]. LIF is frequently overexpressed in different types of human tumors, including breast cancer, colorectal cancer, nasopharyngeal carcinoma, lung cancer and melanoma [3–6]. Overexpression of LIF increases the abilities of migration and invasion in tumor cells *in vitro* and promotes tumor metastasis *in vivo* [3, 7]. Overexpression of LIF in tumors is often associated with poor survival, which substantiates a critical role of LIF in promoting tumor progression and metastasis [3–5]. Despite the important role of LIF in tumor metastasis, its underlying mechanism is far from clear.

Epithelial-mesenchymal transition (EMT) is an evolutionarily conserved and genetically controlled process that allows epithelial cells to acquire mesenchymal features and increased motility and invasiveness [8, 9]. The characteristic of EMT includes the reduced intercellular adhesion, loss of epithelial marker (such as E-cadherin) and acquisition of mesenchymal markers (including Vimentin and N-cadherin) [10]. While EMT is important for normal development, it is also an early and critical step in the metastasis of many epithelial tumors. EMT of cancer cells allows them to leave the primary tumor site, invade and migrate to surrounding and distant regions/organs. Several oncogenic pathways including Ras, Src, integrin, Wnt/β-catenin and Notch signaling have been reported to induce EMT [9, 10]. A number of molecular processes involved in EMT include the activation of transcription factors, the change of expression levels of specific cell surface and cytoskeleton proteins, and the production of some extracellular matrix degradation enzymes. Accumulating evidence suggests that some microRNAs (miRNAs) play important roles in EMT (Table 1). miRNAs are endogenously expressed small RNAs, which regulate gene products at the post-transcriptional level [11]. miRNAs generally bind to 3′ untranslated regions (3′-UTRs) of their target mRNAs to inhibit their translation or promote their degradation [11]. Aberrant expression of miRNAs, including those regulating EMT and cancer metastasis, has been frequently observed in many types of cancers, and plays an important role in the development and progression of tumorigenesis [11–14].

**Table 1:** MicroRNAs involved in EMT

miRNA	Effect on EMT	Reference
Let-7a,b,c,d	Inhibit EMT	[46, 47]
miR-34a	Inhibit EMT	[48]
miR-128-2	Inhibit EMT	[49, 50]
miR-145	Inhibit EMT	[51]
miR-200a,b,c	Inhibit EMT	[47, 52, 53]
miR-203	Inhibit EMT	[54, 55]
miR-214	Inhibit EMT	[56]
miR-21	Promote EMT	[34, 57–59]
miR-106b-25	Promote EMT	[60]
miR-183	Promote EMT	[61]

In this study, we found that LIF promotes EMT in human tumor cells. Overexpression of LIF promoted morphological changes of cells from epithelial-like to mesenchymal-like, increased the expression of mesenchymal markers and decreased an epithelial marker in human cancer cells. Knockdown of endogenous LIF reversed EMT in cancer cells. Furthermore, LIF induced the expression of miR-21, a miRNA that promotes EMT, through its activation of STAT3, a transcription factor that serves as a critical down-stream mediator of LIF. The induction of miR-21 by LIF mediates the promoting effect of LIF on EMT; blocking miR-21 function greatly abolished the promoting effect of LIF on EMT and migration ability in cancer cells. Taken together, results from this study revealed an important function and a novel underlying mechanism of LIF in EMT and tumor metastasis.

## RESULTS

### LIF levels are associated with the levels of EMT markers in human breast cancer cell lines

Our recent report showed that LIF promotes the invasion and migration of *in vitro* cultured breast cancer cells and lung metastasis in nude mice injected with breast cancer cells *via* the tail vein [3]. LIF is frequently overexpressed in many different cancers, including breast cancer [3–6]. Consistent with these previous reports, analysis in 2 different breast cancer datasets from Oncomine (GSE14548 and GSE9014) [15, 16] showed that LIF mRNA levels were elevated in invasive breast carcinomas compared with the normal breast tissues (Supplementary Figure 1).

**Figure 1:**
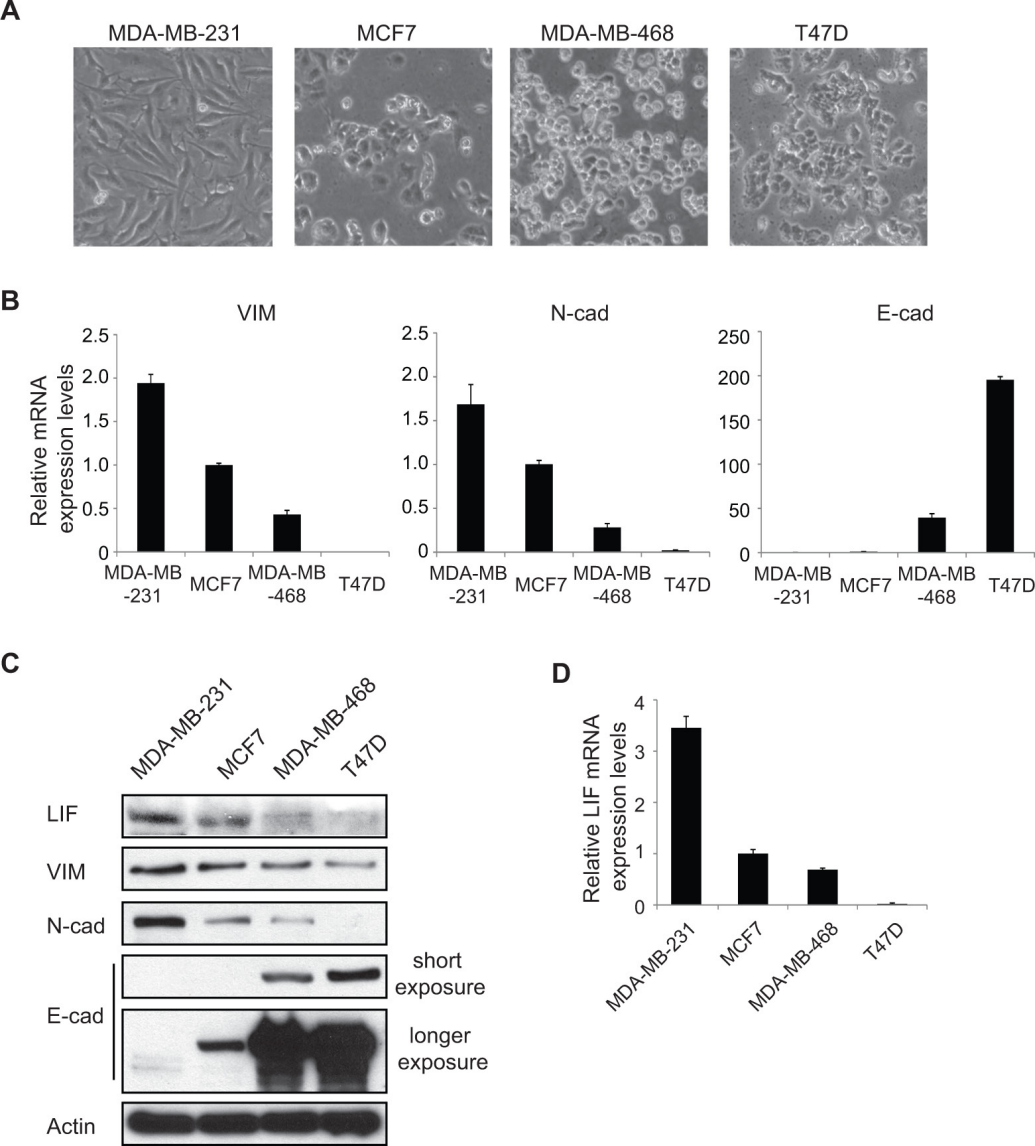
LIF expression levels are associated with the levels of EMT markers in human breast cancer cell lines (**A**) The phase-contrast photomicrographs display the morphology of a group of human breast cancer cell lines (MDA-MB-231, MCF7, MDA-MB-468 and T47D). (**B**) The mRNA expression levels of mesenchymal markers Vimentin (VIM), N-cadherin (N-cad) and epithelial marker E-cadherin (E-cad) were determined in above mentioned breast cancer cell lines by real-time PCR. The mRNA levels of these genes were normalized to β-actin. (**C**) The protein levels of mesenchymal markers (VIM, N-cad) and epithelial marker (E-cad) were measured in cells by Western blot assays with short and longer exposure, respectively. (**D**) The mRNA levels of LIF were determined in cells by real-time PCR. In B and D, data are presented as mean ± s.d. (*n* = 3).

EMT is the initial and critical step of metastasis of many human cancers. To investigate whether LIF plays any role in EMT, we examined the expression levels of LIF in a group of human breast cancer cell lines with different EMT status, including MDA-MB-231, MCF7, MDA-MB-468 and T47D cells. Among these cell lines, MDA-MB-231 cells are more mesenchymal-like with a higher metastatic ability, whereas MCF7, MDA-MB-468 and T47D cells are more epithelial-like with a lower metastatic ability [17] (Figure 1A). Consistent with the morphology of these cells, MDA-MB-231 cells express much higher levels of mesenchymal markers Vimentin and N-cadherin and express much lower levels of epithelial marker E-cadherin compared with the other 3 cell lines at both mRNA and protein levels as determined by real-time PCR and Western blot assays, respectively (Figure 1B and 1C). Interestingly, LIF expression at both mRNA and protein levels are much higher in MDA-MB-231 cells compared with MCF7, MDA-MB-468 and T47D cells (Figure 1C and 1D). These results suggest a positive correlation of LIF expression levels with the mesenchymal markers Vimentin and N-cadherin, and a negative correlation of LIF expression levels with the epithelial marker E-cadherin, which raised the possibility that LIF may play an important role in EMT in human cancers.

### Ectopic expression of LIF promotes EMT of human cancer cells

**Figure 2:**
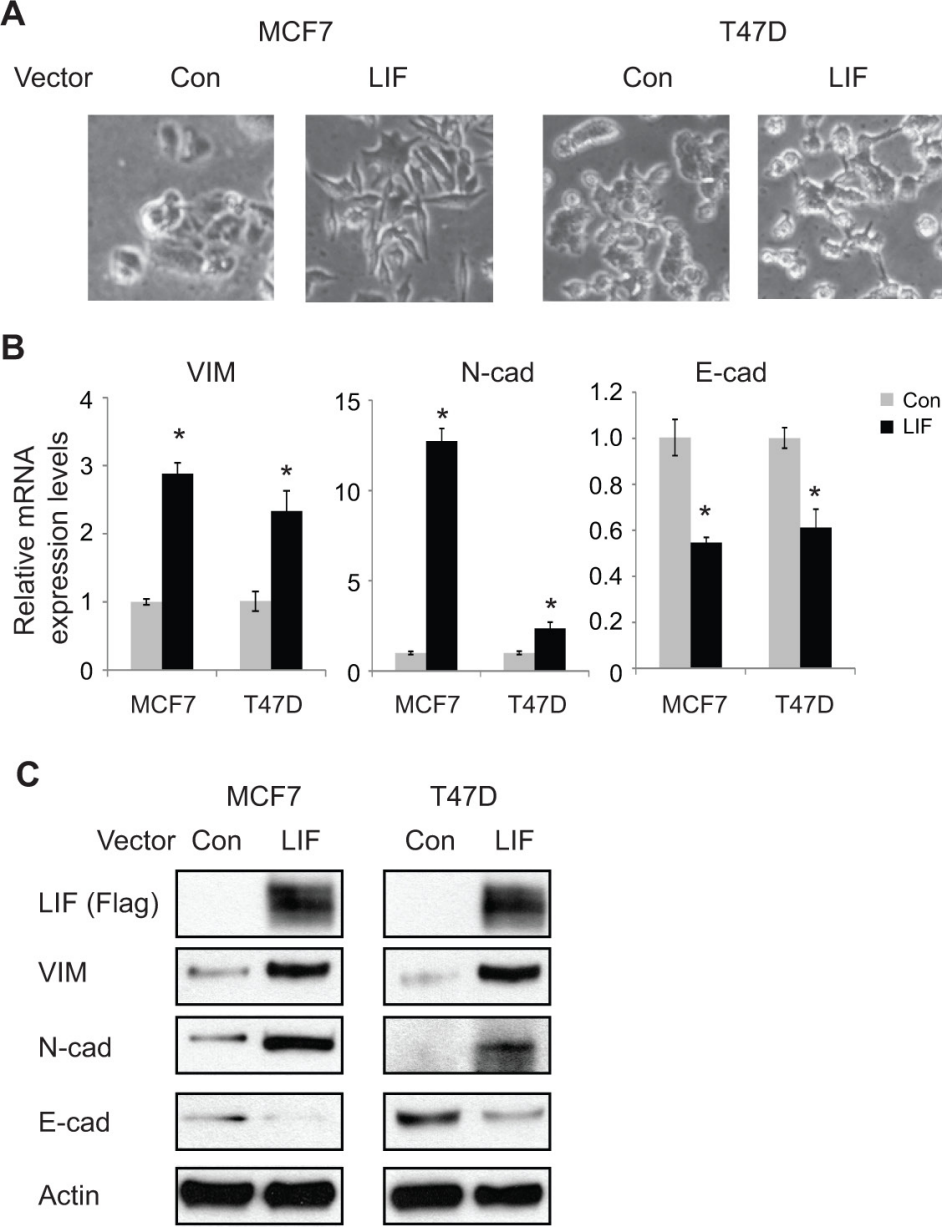
Ectopic expression of LIF induces EMT in MCF7 and T47D cells (**A**) MCF7 and T47D cells were transiently transfected with LIF expression vectors or control vectors (Con). EMT morphological change was examined by phase-contrast photomicrographs at 48 h after transfection. (**B**) The mRNA expression levels of mesenchymal markers (VIM, N-cad) and epithelial marker (E-cad) were determined in cells at 48 h after transfection of LIF expression vectors or control vectors by real-time PCR. The mRNA levels of these genes were normalized to β-actin. Data are presented as mean ± s.d. (*n* = 3). *p < 0.05; student *t*-test. (**C**) The protein levels of mesenchymal markers (VIM, N-cad) and epithelial marker (E-cad) were measured in cells by Western blot assays.

To investigate the potential role of LIF in EMT, we employed MCF7 and T47D cells with transient ectopic expression of LIF by transient transfection of LIF expression vectors and their control cells transfected with control vectors. Both of these cell lines have relatively low levels of endogenous LIF and have epithelial-like morphology (Figure 1A–1C). Ectopic LIF expression clearly induced the classic morphological changes commonly associated with EMT in both cell lines (Figure 2A). Consistent with the morphological changes induced by LIF, ectopic LIF expression greatly increased the expression of mesenchymal markers including Vimentin and N-cadherin, and decreased the expression of epithelial marker E-cadherin at both mRNA and protein levels as determined by real-time PCR and Western blot assays, respectively (Figure 2B and 2C). Similar results were observed in both cells with stable ectopic LIF expression; stable ectopic LIF expression induced classic morphological EMT changes and changed the expression of EMT markers including Vimentin, N-cadherin and E-cadherin at both mRNA and protein levels (Supplementary Figure 2A–2C). Consistent with these results obtained from breast cancer cell lines, transient transfection of LIF to human colorectal cancer cell line HCT116 clearly increased the mRNA expression levels of mesenchymal markers (Vimentin and N-cadherin) and decreased the expression levels of epithelial marker E-cadherin (Supplementary Figure 3), suggesting that LIF promotes EMT in different types of tumors.

**Figure 3:**
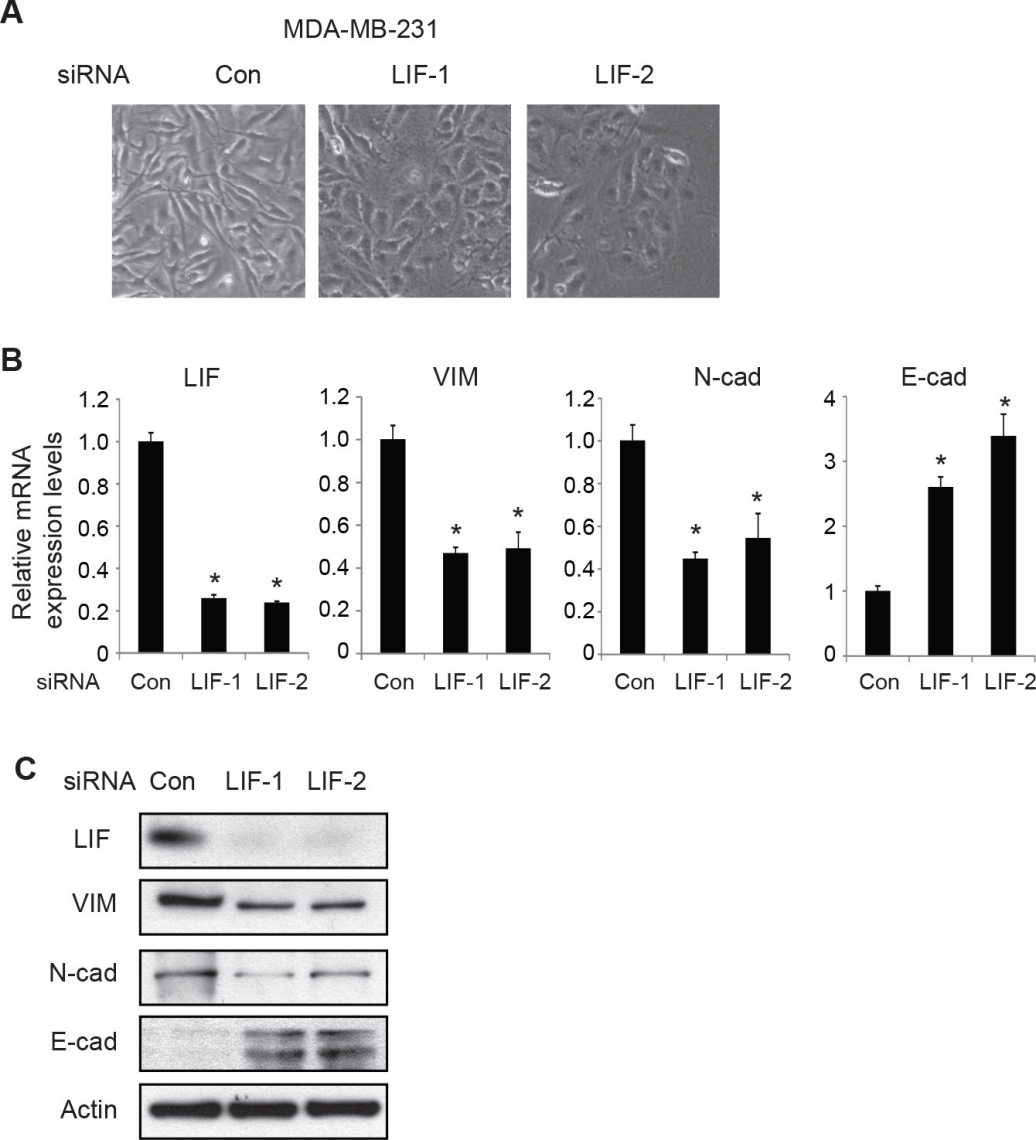
Knockdown of endogenous LIF reverses EMT in MDA-MB-231 cells (**A**) MDA-MB-231 cells were transfected with control siRNA (con) or two different siRNA oligos targeting LIF. EMT morphological change was examined by phase-contrast photomicrographs. (**B**) Knockdown of endogenous LIF changed the mRNA expression levels of EMT markers in MDA-MB-231 cells as determined by real-time PCR. The mRNA levels of these genes were normalized to β-actin. Data are presented as mean ± s.d. (*n* = 3). *p < 0.05; student *t*-test. (**C**) The protein levels of EMT markers were measured in cells by Western blot assays.

We further examined the effect of knocking down endogenous LIF on EMT by employing MDA-MB-231 cells which have high endogenous LIF levels. Knockdown of endogenous LIF by two different siRNA oligos in MDA-MB-231 cells induced morphological changes of cells from mesenchymal-like to epithelial-like (Figure 3A). Furthermore, knockdown of LIF decreased the expression levels of mesenchymal markers Vimentin and N-cadherin and increased the expression levels of epithelial marker E-cadherin at both mRNA and protein levels (Figure 3B and 3C). Similar results were obtained in MDA-MB-231 cells with stable LIF knockdown by using 2 different shRNA vectors against LIF (Supplementary Figure 4). These results demonstrate that LIF promotes EMT of human cancer cells.

### LIF promotes EMT through the induction of miR-21 expression

**Figure 4:**
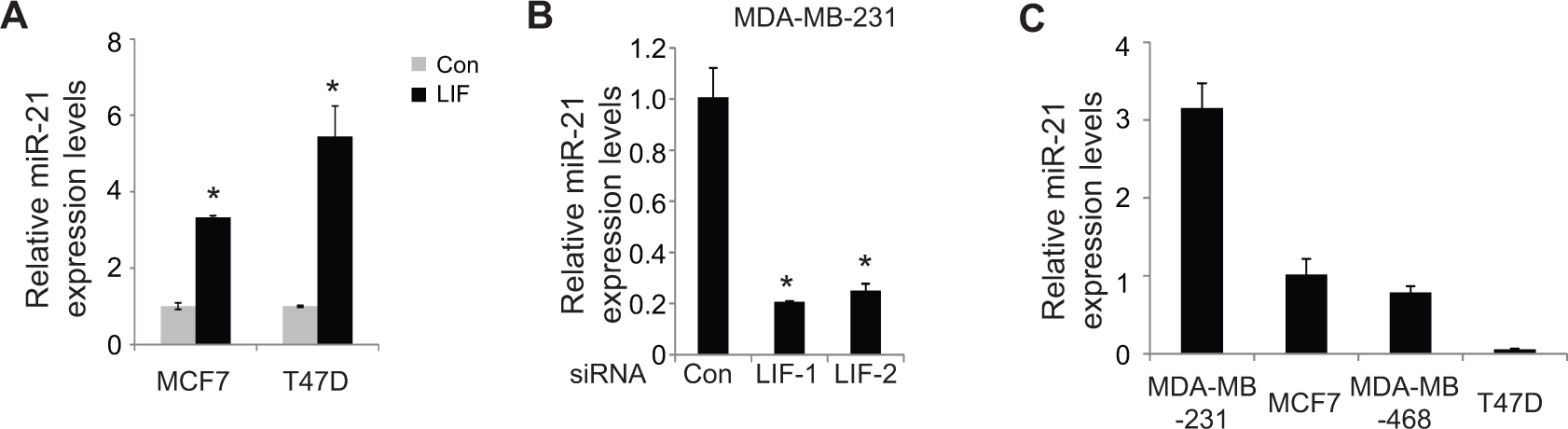
LIF induces the expression of miR-21 (**A**) Ectopic expression of LIF induced miR-21 expression levels in MCF7 and T47D cells as determined by real-time PCR. The expression of miR-21 was normalized to the U6 snRNA. (**B**) Knockdown of endogenous LIF by two siRNA oligos reduced miR-21 expression levels in MDA-MB-231 cells. (**C**) The expression levels of miR-21 in MDA-MB-231, MCF7, MDA-MB-468 and T47D cells. Data are presented as mean ± s.d. (*n* = 3). *p < 0.05, student *t*-test.

miRNAs are a group of small non-coding RNA molecules, which regulate gene expression by targeting mRNAs to induce either translation repression or degradation of targeting mRNAs [11]. Recent studies have shown that miRNAs can function as either oncogenes or tumor suppressors to play critical roles in the regulation of cancer progression, including EMT [12, 14, 18, 19]. LIF can transcriptionally regulate some genes through the activation of several signaling pathways, including STAT3. The regulation of miRNAs by LIF is poorly understood. Here, we investigated the effect of LIF on the expression levels of a group of miRNAs that are involved in EMT as listed in Table 1 in MCF7 and T47D cells with and without ectopic LIF expression by using real-time PCR assays. Among these miRNAs, LIF showed strong induction of miR-21 in both cell lines (Figure 4A). The induction of miR-21 by LIF was also observed in MCF7 and T47D cells with stable ectopic expression of LIF and HCT116 cells transfected with LIF expression vectors (Supplementary Figure 5A and 5B). Furthermore, knockdown of endogenous LIF by siRNA clearly decreased miR-21 expression levels in MDA-MB-231 cells (Figure 4B). Similar results were obtained in MDA-MB-231 cells with stable endogenous LIF knockdown by shRNA vectors (Supplementary Figure 5C). Notably, the expression levels of miR-21 also correlate with LIF expression levels in a panel of breast cancer cell lines with different EMT status. The expression levels of miR-21 are much higher in MDA-MB-231 cells with higher endogenous LIF levels and higher EMT potential than in MCF7, MDA-MB-468 and T47D cells which have lower basal LIF expression levels and lower EMT potential (Figure 4C).

**Figure 5:**
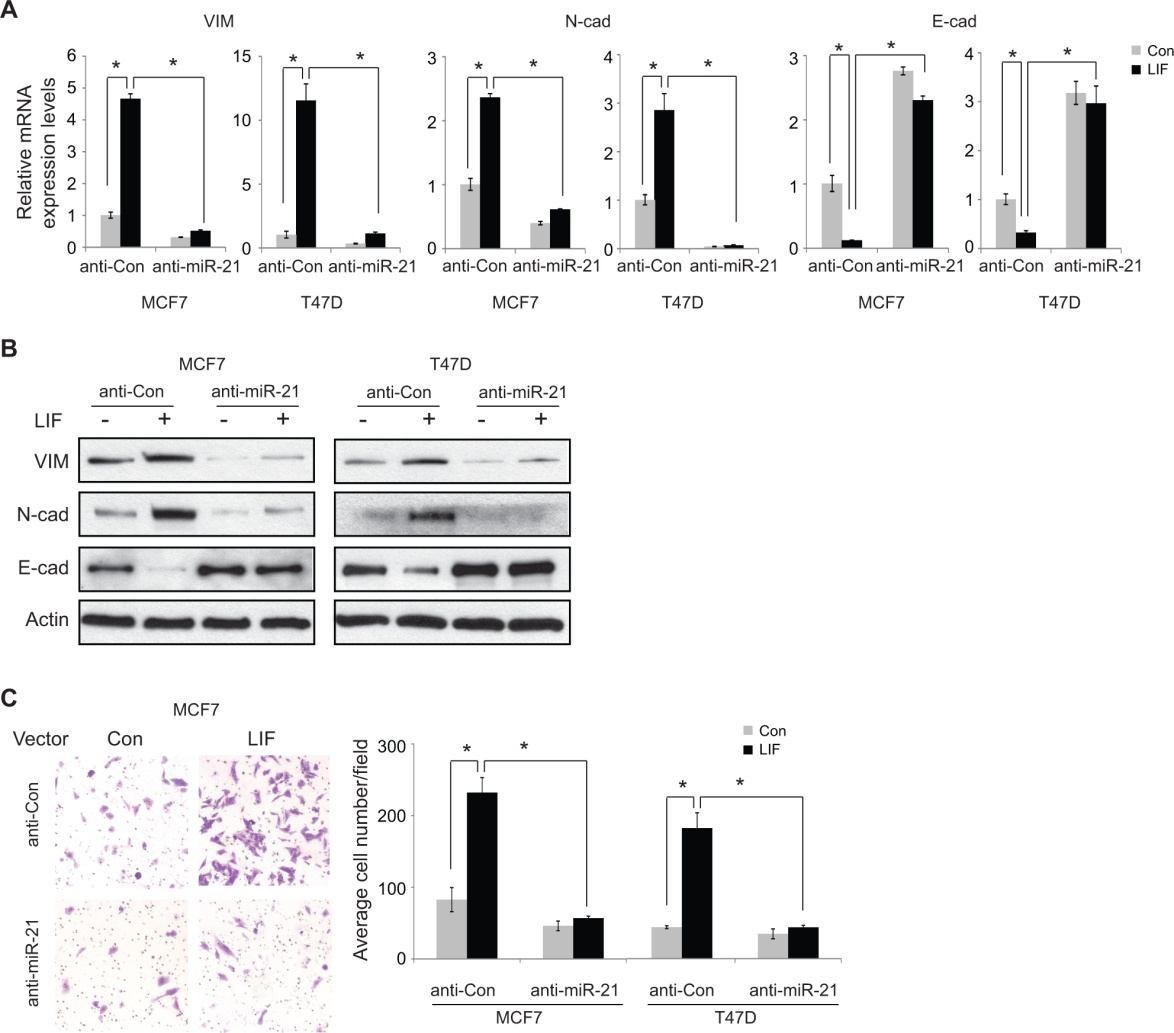
Blocking miR-21 largely abolishes the effect of LIF on inducing EMT (**A** and **B**) MCF7 and T47D cells with and without stable ectopic expression of LIF were transfected with anti-miR-21 or control RNA oligos. The mRNA (A) and protein (B) levels of EMT markers were determined by real-time PCR and Western blot assays, respectively. The mRNA levels of these genes were normalized to β-actin. Data are presented as mean ± s.d. (*n* = 3). (**C**) Blocking miR-21 largely abolished the promoting effect of LIF on migration ability in MCF7 and T47D cells. MCF7 and T47D cells with and without stable ectopic LIF expression were transfected with anti-miR-21 or control RNA oligos. The migration ability of cells was determined by trans-well assays. Left panels: representative images; right panels: quantification of average number of migrated cells/field. *p < 0.05; student *t*-test.

The effect of miR-21 on EMT was examined in MCF7 cells. Consistent with previous reports [20–22], overexpression of miR-21 by transfection of miR-21 mimics clearly increased the expression of mesenchymal markers Vimentin and N-cadherin and decreased the expression of epithelial marker E-cadherin at both mRNA and protein levels as determined by real-time PCR and Western blot assays, respectively (Supplementary Figure 6). To investigate whether the induction of miR-21 by LIF contributes to the promoting effect of LIF on EMT, miR-21 was blocked by anti-miR-21 oligos in MCF7 and T47D cells with and without ectopic LIF expression, and the effect of LIF on the expression of EMT markers was determined. Blocking miR-21 decreased the expression of mesenchymal markers Vimentin and N-cadherin and increased the expression of epithelial marker E-cadherin in both cell lines (Figure 5A and 5B). Notably, anti-miR-21 oligos largely inhibited the effect of LIF on the expression of EMT markers, including mesenchymal markers (Vimentin and N-cadherin) and epithelial marker (E-cadherin) in both cell lines. In cells transfected with anti-miR-21 oligos, ectopic expression of LIF showed no apparent effect on the expression of EMT markers (Figure 5A and 5B). Our previous study reported that LIF promotes the migration ability of tumor cells [3]. Blocking miR-21 by anti-miR-21 oligos clearly inhibited the migration ability of MCF7 and T47D cells. Importantly, blocking miR-21 largely abolished the promoting effect of LIF on cell migration ability (Figure 5C). Together, these results strongly suggest that LIF promotes EMT of cancer cells through the induction of miR-21.

### LIF upregulates miR-21 through the STAT3 signal pathway

**Figure 6:**
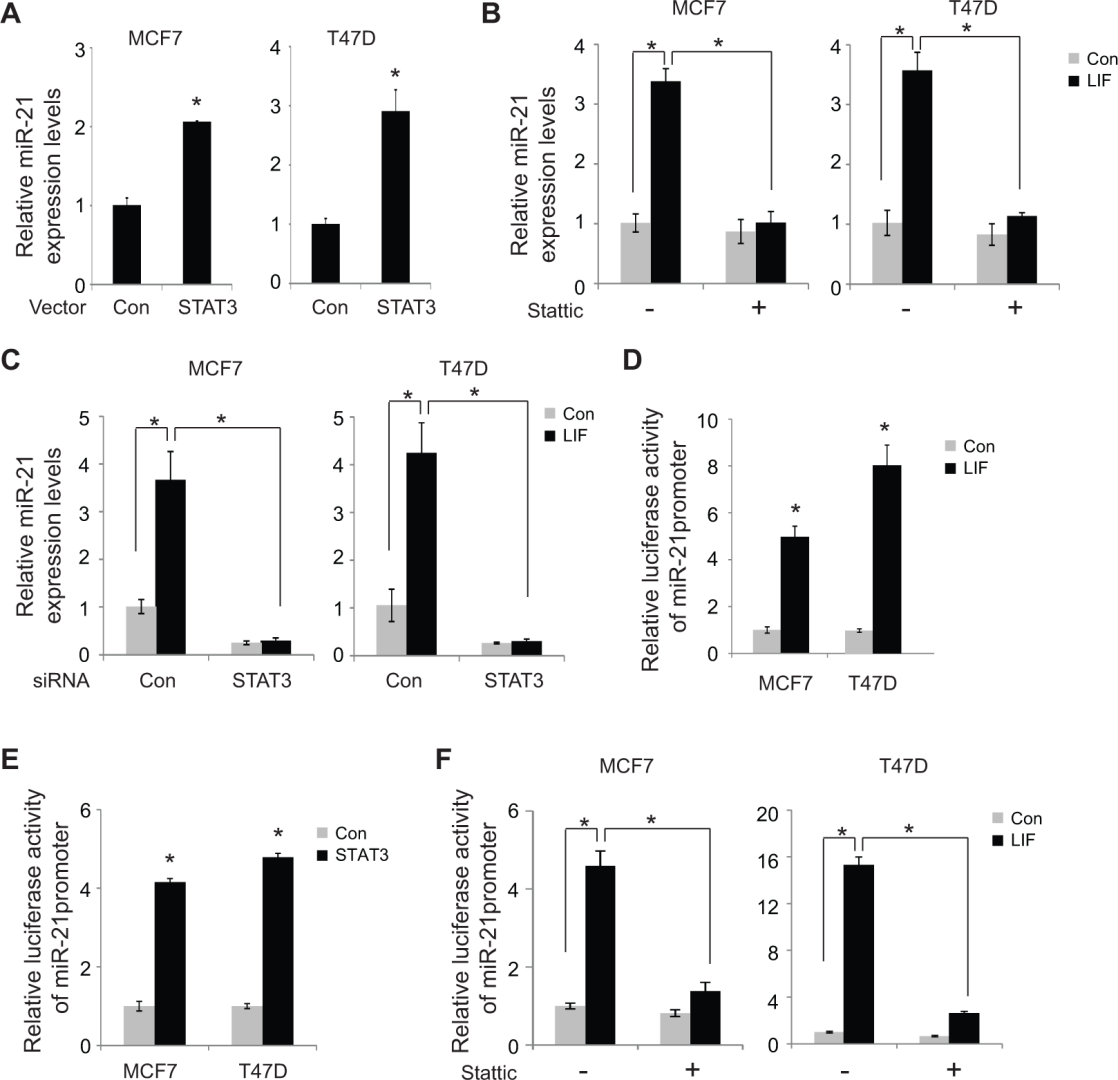
LIF induces miR-21 expression through STAT3 (**A**) MCF7 and T47D cells were transfected with STAT3 expression vectors or control vectors. The expression levels of miR-21 were determined at 24 h after transfection by real-time PCR. The expression of miR-21 was normalized to the U6 snRNA. (**B**) Stattic treatment largely abolished the induction of miR-21 by LIF. MCF7 and T47D cells with stable ectopic LIF expression and their control cells were treated with Stattic (2 μM), and the miR-21 expression levels were determined by real-time PCR. (**C**) Knockdown of endogenous STAT3 by siRNA oligos largely abolished the induction of miR-21 by LIF. MCF7 and T47D cells with stable ectopic expression of LIF and their control cells were transfected with siRNA oligos targeting STAT3 or control siRNA. The expression levels of miR-21 were determined using real-time PCR. (**D**) MCF7 and T47D cells with or without ectopic LIF expression were transfected with the luciferase reporter vectors containing the miR-21 promoter together with pRL-null vectors as an internal control to normalize transfection followed by luciferase activity measurement. The luciferase activities of reporter vectors were normalized to internal control. (**E**) MCF7 and T47D cells were transfected with the luciferase reporter vectors containing the miR-21 promoter together with pRL-null vectors and STAT3 expression vectors. (**F**) MCF7 and T47D cells transfected with luciferase reporter vectors together with LIF expression vectors or control vectors were treated with Stattic (2 μM). Luciferase activities were measured after 24 h of treatment. Data are presented as mean ± s.d. (*n* = 3). *p < 0.05, student *t*-test.

STAT3 is a transcription factor and a critical downstream effector of the LIF signaling. LIF can phosphorylate and activate STAT3, which in turn transcriptionally induces a group of down-stream target genes to mediate many biological functions of LIF [23, 24]. To investigate whether LIF induces miR-21 expression through STAT3, the effect of STAT3 on miR-21 expression was examined in MCF7 and T47D cells. Overexpression of STAT3 by transfection of STAT3 expression vectors clearly induced the expression of miR-21 in both MCF7 and T47D cells (Figure 6A). To investigate whether STAT3 mediates the induction of miR-21 by LIF, Stattic, a specific STAT3 inhibitor, was employed to block STAT3 function, and the effect of LIF on miR-21 expression was examined in both MCF7 and T47D cells. As shown in Figure 6B, Stattic largely abolished the induction of miR-21 by LIF in both cells. Similar results were obtained when STAT3 function was inhibited by knocking down endogenous STAT3 using siRNA targeting STAT3 (Figure 6C). The transcriptional activation of miR-21 by LIF through STAT3 was further determined by luciferase reporter assays. MCF7 and T47D cells with or without ectopic LIF expression were transfected with the luciferase reporter vector (pGL2) containing the promoter region of miR-21. As shown in Figure 6D, LIF clearly increased luciferase activity of the reporter vector containing miR-21 promoter. Similarly, ectopic overexpression of STAT3 by transient transfection of STAT3 expression vectors clearly increased luciferase activity of the vector containing miR-21 promoter in MCF7 and T47D cells (Figure 6E). Notably, blocking STAT3 function using Stattic largely abolished the effect of LIF on inducing the luciferase activity of miR-21 reporter in MCF7 and T47D cells (Figure 6F), which strongly suggests that LIF induces miR-21 expression through STAT3.

**Figure 7:**
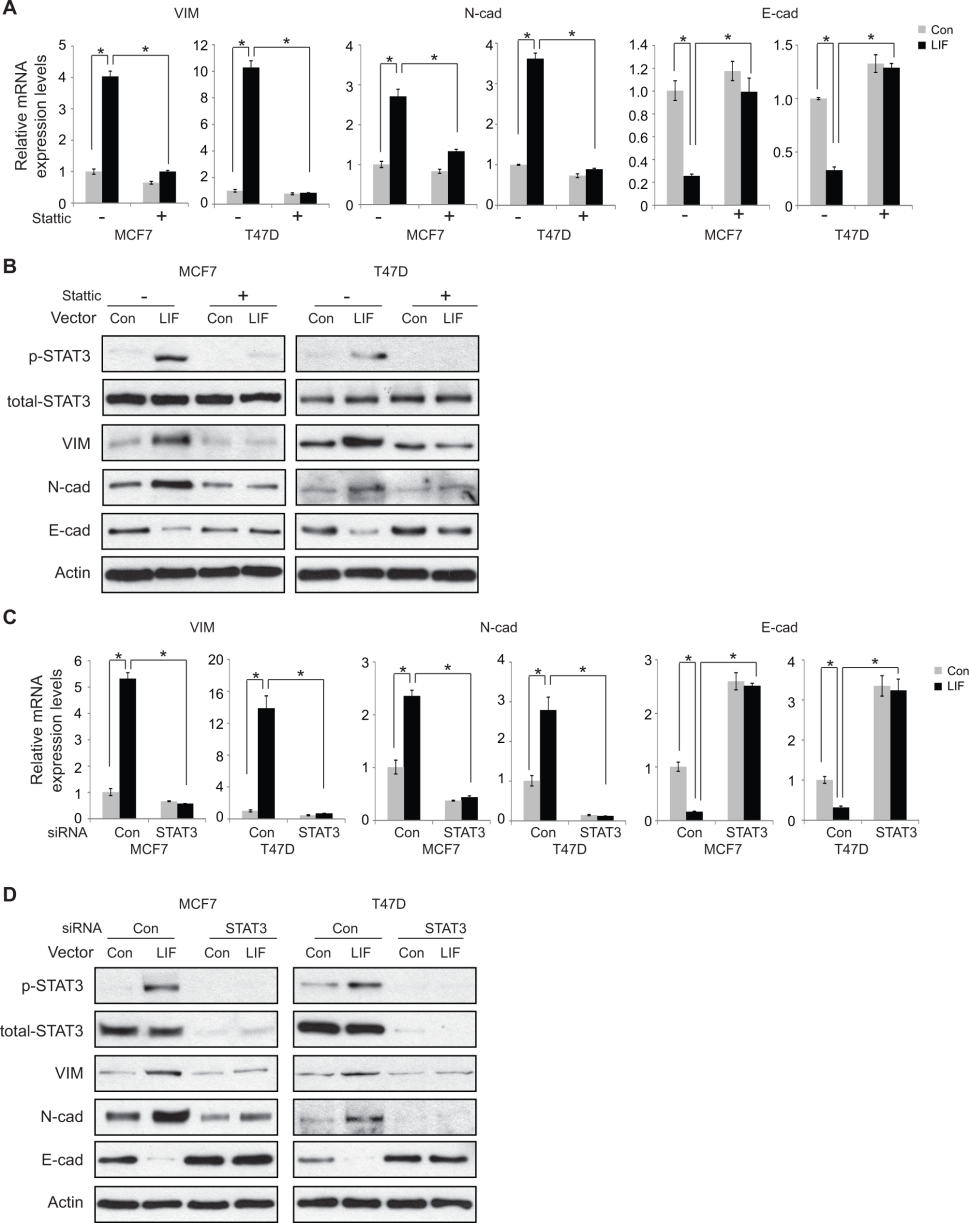
STAT3 mediates the promoting effect of LIF on EMT in MCF7 and T47D cells (**A** and **B**) Blocking STAT3 function by Stattic largely abolished the effect of LIF on the expression of EMT markers. MCF7 and T47D cells with ectopic stable LIF expression were treated with Stattic (2 μM) for 24 h. The expression levels of EMT markers were determined at both mRNA (A) and protein (B) levels by real-time PCR and Western blot assays, respectively. (**C** and **D**) Knockdown of endogenous STAT3 by siRNA largely abolished the effect of LIF on the expression of EMT markers. MCF7 and T47D cells with ectopic stable LIF expression were transfected with siRNA targeting STAT3 or control siRNA. The expression levels of EMT markers were determined at both mRNA (C) and protein (D) levels by real-time PCR and Western blot assays, respectively. The levels of total STAT3 and phosphorylated STAT3 at Tyrosine 705 (p-STAT3) were determined by Western blot assays (B and D). For A and C, data are presented as mean ± s.d. (*n* = 3). *p < 0.05; student *t*-test.

We further examined whether STAT3, which induces miR-21, mediates the effect of LIF on EMT. Ectopic STAT3 expression clearly increased the mRNA expression of mesenchymal markers including Vimentin and N-cadherin, and decreased the expression of epithelial marker E-cadherin in MCF7 and T47D cells (Supplementary Figure 7), suggesting that STAT3 promotes EMT in cancer cells. These results are consistent with previous reports [25, 26]. Importantly, blocking STAT3 function by Stattic largely decreased the effect of LIF on the expression of EMT markers at both mRNA and protein levels in MCF7 and T47D cells (Figure 7A and 7B). The effective blocking of STAT3 signaling by Stattic is confirmed by the loss of phosphorylation of STAT3 in cells treated with LIF (Figure 7B). Consistent results were obtained in both MCF7 and T47D cells when STAT3 function was blocked by using siRNA oligos targeting STAT3. Knockdown of endogenous STAT3 by siRNA oligos greatly reduced the effect of LIF on the expression of EMT markers at both mRNA and protein levels in MCF7 and T47D cells (Figure 7C and 7D). Together, these results strongly suggest that LIF upregulates miR-21 through the STAT3 signaling pathway to promote EMT in cancer cells.

## DISCUSSION

LIF has a complex role in cancer. Early work on LIF revealed its function in inducing the differentiation of myeloid leukemia cells [27]. That is how LIF got its name as leukemia inhibitory factor. Recent studies including ours demonstrate that LIF promotes the development and progression of solid tumors [3–5]. LIF overexpression increases the proliferation rate of *in vitro* cultured cancer cells, the growth rate of xenograft tumors and metastasis of many different types of human tumors [3–7]. A recent report shows that LIF promotes proinvasive activation of carcinoma-associated stromal fibroblasts, which results in increased cancer cell invasion [28]. In addition, LIF overexpression promotes resistance towards chemotherapy and radiotherapy [4, 5]. LIF overexpression is frequently observed in many human tumors, including breast cancer, colorectal cancer, lung cancer, head and neck cancer, melanoma and nasopharyngeal carcinoma. Patients with higher levels of LIF in tumors often have poor clinical outcomes [3–7]. These observations strongly suggest that LIF is an important factor in promoting tumorigenesis, especially in solid tumors.

While accumulating evidence supports the role of LIF in different types of tumors, its underlying molecular mechanisms are poorly understood. LIF can activate several signaling pathways, including STAT3, AKT, MAPK and mTOR, in a cell and tissue-type specific manner. Many of these signaling pathways are aberrantly activated in tumors and play important roles in tumorigenesis. Our recent study reports that LIF can down-regulate the levels and function of p53, a central player for tumor suppression, in colorectal cancer cells [5]. In addition to its regulatory role in tumor cells, LIF promotes invasive tumor microenvironment through the activation of stromal fibroblast, which could be an important mechanism for the promoting effect of LIF on tumor invasion and metastasis [28].

Results from this study show that LIF promotes EMT of tumor cells, which is a new and important mechanism by which LIF promotes cancer metastasis. Overexpression of LIF in breast cancer and colorectal cancer cells promotes cancer cells to acquire mesenchymal features, increase the expression of mesenchymal markers, including Vimentin and N-cadherin, and decrease the expression of E-cadherin. In turn, LIF overexpression clearly increases the migration and invasion abilities of cancer cells (Figure 5C) [3]. miRNAs are an important group of regulators for EMT. Among them, miR-21 has been shown to play an important role in promoting EMT and metastasis [20–22]. miR-21 increases the expression of Vimentin and N-cadherin and decreases the expression of E-cadherin (Supplementary Figure 6). miR-21 has been shown to target multiple genes, including PTEN, TIAM1, PDCD4 and maspin [31–34]. The products of these genes are all involved in the inhibition of migration, invasion and metastasis. Overexpression of miR-21 is frequently observed in many human tumors, including breast cancer, colorectal cancer, lung cancer and pancreatic cancer. High miR-21 levels are correlated with advanced clinic stage and lymph node metastasis in breast cancer [29, 30]. Furthermore, high expression levels of miR-21 in tumors are often associated with poor survival of patients with breast cancer, lung cancer, colorectal cancer and pancreatic cancer [29, 35–37]. Results from this study show that LIF induces the expression of miR-21 through the STAT3 signaling in human tumor cells. Blocking the function of STAT3 by a STAT3-specific inhibitor largely abolished the induction of miR-21 expression by LIF. Importantly, miR-21 mediates the promoting effect of LIF on EMT and metastasis. Knockdown of endogenous miR-21 clearly abolished the promoting effect of LIF on EMT as well as the migration ability of tumor cells. These results strongly suggest the important role of LIF/STAT3/miR21 in EMT and tumor metastasis.

Recent reports have indicated that EMT promotes the emergence of cancer stem cells (CSCs) [38, 39]. CSCs and the cancer cells with CSC features are often resistant to chemotherapy [40]. It is possible that the induction of EMT by LIF can lead to the resistance towards therapy in cancer patients with increased LIF expression in tumors. Indeed, recent studies including ours show that LIF overexpression enhances resistance towards cancer therapy [4, 5].

miR-21 has been reported to promote the proliferation and growth of tumor cells, a phenotype that is also observed in cells with LIF overexpression [3, 31]. One of its mechanisms is through targeting PTEN by miR-21 to activate AKT pathway (Supplementary Figure 8A) [41, 42]. Our recent study showed that LIF activates the AKT/mTOR signaling in tumor cells [3]. Blocking miR-21 by anti-miR-21 oligos greatly inhibited the activation the AKT/mTOR signaling by LIF as reflected by the phosphorylation levels of AKT and p70S6K (Supplementary Figure 8B). It is possible that the induction of miR-21 by LIF may also contribute to the promoting effect of LIF on cell proliferation and tumor growth, which will be of interest to investigate in future study.

In addition to its role in cancer, EMT is also involved in normal physiological process of embryonic implantation and the initiation of placenta formation. LIF is highly induced at the implantation stage in uterine tissues and regulates several important steps during implantation, including the receptive state of endometrial, the interaction between endometrial and embryo, stromal decidualization, the invasion and development of blastocyst [43]. It is unclear whether the induction of miR-21 by LIF also occurs in uterine tissues during implantation and whether LIF regulates EMT during implantation through miR-21. Future studies will shed some lights on this set of interesting questions.

In summary, this study demonstrates that LIF promotes EMT, which is a novel mechanism by which LIF promotes tumor progression and metastasis. This function of LIF is mainly mediated through the induction of miR-21 by STAT3. Results from this study suggest that targeting LIF/STAT3/miR-21 could be a potential therapeutic strategy for tumors with LIF overexpression.

## MATERIALS AND METHODS

### Cells and reagents

Human breast cancer cell lines MCF7, T47D, MDA-MB-231 and MDA-MB-468 and human colorectal cancer cell line HCT116 were purchased from ATCC. MCF7 and T47D cells were cultured in RPMI1640 with 10% FBS. MDA-MB-231, MDA-MB-468 and HCT116 cells were culture in DMEM with 10% FBS. The ectopic LIF stable cell lines MCF7-LIF and T47D-LIF and MDA-MB-231 cells with stable knockdown of endogenous LIF (MDA-MB-231-shLIF1 and MDA-MB-231-shLIF2) were established in our lab as previously described [3]. Stattic was purchased from Sigma. Recombinant human LIF protein was purchased from Millipore.

The LIF expression vector pLPCX-LIF was constructed in our lab as previously described [3]. The sequence of the primer set used to amplify the promoter/enhancer region of miR-21 is as following: 5′-TTT GGT ACC TTG CTA ATG CAT TCT-3′ and 5′-TTT AGA TCT AGT TCA GCT ATG GTA AGA GC-3′. The amplified miR-21 promoter/enhancer region, which extends from −1120 to +25bp relative to miR-21 transcriptional start site [44], was inserted into pGL2-Basic vector at the Kpn I and Bgl II sites (Promega). The STAT3 expression vector was kindly provided by Dr L. Resar at Johns Hopkins University. Retrovirus shRNAs vectors against human LIF were purchased from Open Biosystems (Thermo Scientific). The plasmids were transfected into the cells using Lipofectamine 2000 (Invitrogen). STAT3 siRNA oligos, LIF siRNA oligos and anti-miR-21oligos were purchased from IDT and transfected into the cells using Oligofectamine (Invitrogen). The sequence of anti-miR-21 oligos is as following: 5′-UCA ACA UCA GUC UGA UAA GCU A-3′ [45].

### Real-time PCR assay

Total miRNA and RNA were extracted from cells by using a miRNesay Mini Kit and an RNeasy Kit, respectively (Qiagen). cDNA was prepared by using a Taqman Reverse Transcription Kit (Life technology). The U6 and miR-21 probes were purchased from Life technology. The expression levels of EMT markers were detected by following primers: E-cadherin (E-cad) forward: 5′-ATT TTT CCC TCG ACA CCC GAT-3′, reverse: 5′-TCC CAG GCG TAG ACC AAG A-3′, N-cadherin (N-cad) forward: 5′-TGC GGT ACA GTG TAA CTG GG-3′, reverse: 5′-GAA ACC GGG CTA TCT GCT CG-3′ and Vimentin (VIM) forward: 5′-AAT GGC TCG TCA CCT TCG TGA AT-3′, reverse: 5′-CAG ATT AGT TTC CCT CAG GTT CAG-3′.

### Western blot assays

Standard Western blot assays were used to analyze the levels of protein. The EMT markers were detected by the following antibodies: anti-E-cad (sc-7870, 1:2000, Santa Cruz), anti-N-cad (sc-393933, 1:2000, Santa Cruz) and anti-VIM (sc-7558, 1:1000, Santa Cruz). LIF was detected by anti-LIF (AF-250-NA, 1:1000, R & D). Anti-p-STAT3 (9145L, 1:1000, Cell signaling) and anti-STAT3 (sc-482, 1:2000, Santa Cruz) antibodies were used to detect the activation of the STAT3 signaling pathway.

### Migration assays

For migration assays, the trans-well system (24 wells, 8 μM pore size, BD Biosciences) was employed as we previously described [3]. In brief, cells transfected with anti-miR21 or control oligos were seeded into the upper chambers in FBS free medium, and the lower chambers were filled with culture medium containing FBS. Cells on the lower surface were fixed, stained and counted after culturing for 24 or 48 h. Image J software was used to calculate the cell numbers in the captured fields.

### Luciferase activity assay

Cells were transiently transfected with luciferase report vector pGL2 containing miR-21 promoter region together with pRL-null vector which expresses *Renilla* as an internal control to normalize transfection efficiency by using Lipofectamine 2000 (Invitrogen). The luciferase activity was measured by using the Luciferase Assay Kit (Promega) and normalized with *Renilla* luciferase activity. To determine whether STAT3 transactivates the pGL2-miR-21 reporter vector, cells were co-transfected with pGL2-miR-21 reporter vector, pRL-null vector and STAT3 expression vector.

### Statistical analysis

The Student *t*-test was used to identify the difference between two groups. Values of *p* < 0.05 were considered to be statistically significant.

## SUPPLEMENTARY MATERIALS FIGURES


