# Supplementary material for: Leukemia inhibitory factor promotes EMT through STAT3-dependent miR-21 induction
Source: Oncotarget. 2015 Dec 24;7(4):3777–90. doi: 10.18632/oncotarget.6756 (PMC4826169; doi:10.18632/oncotarget.6756)
Supplement: Supplementary file 1 [file oncotarget-07-3777-s001.pdf]

Leukemia inhibitory factor promotes EMT through STAT3-dependent miR-21 induction

Supplementary Materials

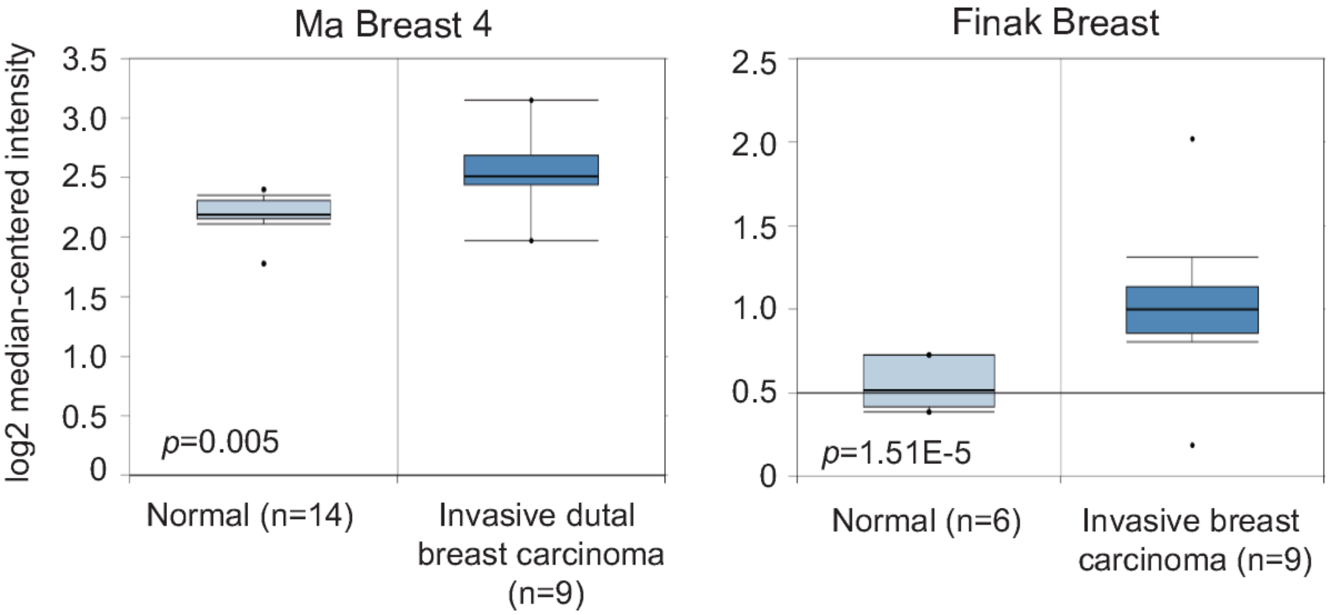

Supplementary Figure S1: LIF mRNA levels are frequently elevated in tumors. LIF mRNA levels are elevated in invasive breast carcinomas in 2 different datasets from the Oncomine database (GSE14548 and GSE9014). LIF mRNA levels are presented as box plots and expressed in terms of a log2 median-centered intensity which is calculated by normalizing the intensity of LIF probe to the median of the probe intensities across the entire array [15, 16].

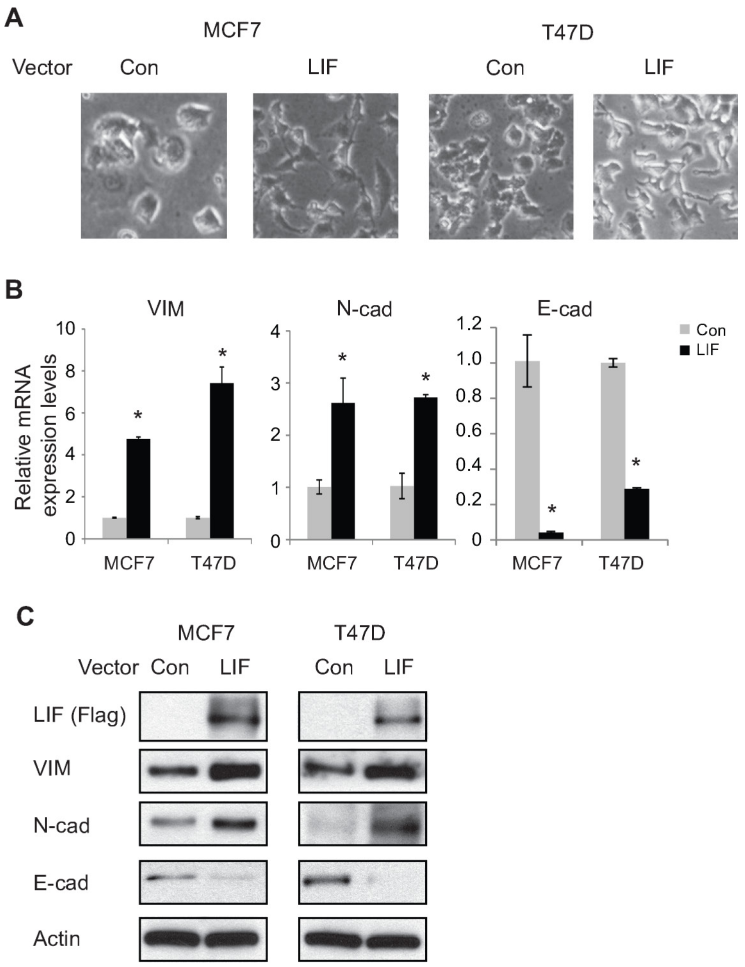

Supplementary Figure S2: Stable ectopic LIF expression promotes EMT in MCF7 and T47D cells. (**A**) EMT morphological change was examined by phase-contrast photomicrographs in MCF7 and T47D cells with stable ectopic LIF expression and control cells stably transfected with control expression vectors (Con). (**B and C**) The mRNA (B) and protein (C) levels of mesenchymal markers (VIM, N-cad) and epithelial marker (E-cad) were determined in cells by real-time PCR and Western blot assays, respectively. The mRNA levels of these genes were normalized to β- actin. Data are presented as mean ± s.d. (n = 3). *$p < 0.05$; student t-test.

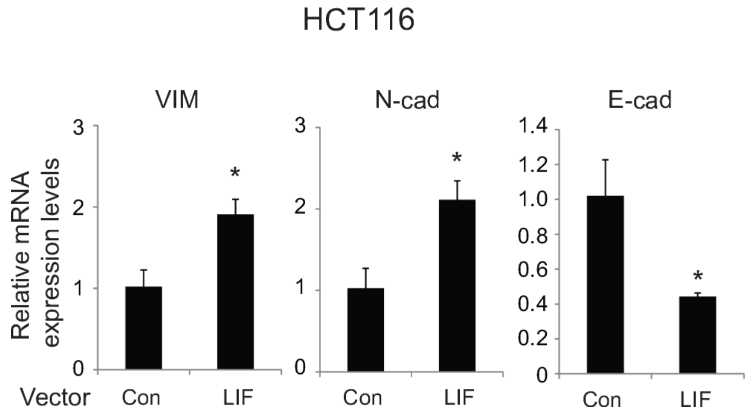

Supplementary Figure S3: Ectopic LIF expression changes the expression levels of EMT markers in human colorectal cancer HCT116 cells. HCT116 cells were transiently transfected with LIF expression vectors or control vectors (Con). The mRNA expression levels of mesenchymal markers (VIM, N-cad) and epithelial marker (E-cad) were determined in cells at 48 h after transfection by real-time PCR. The mRNA levels of these genes were normalized to β-actin. Data are presented as mean ± s.d. (n = 3). *$p < 0.05$; student t-test.

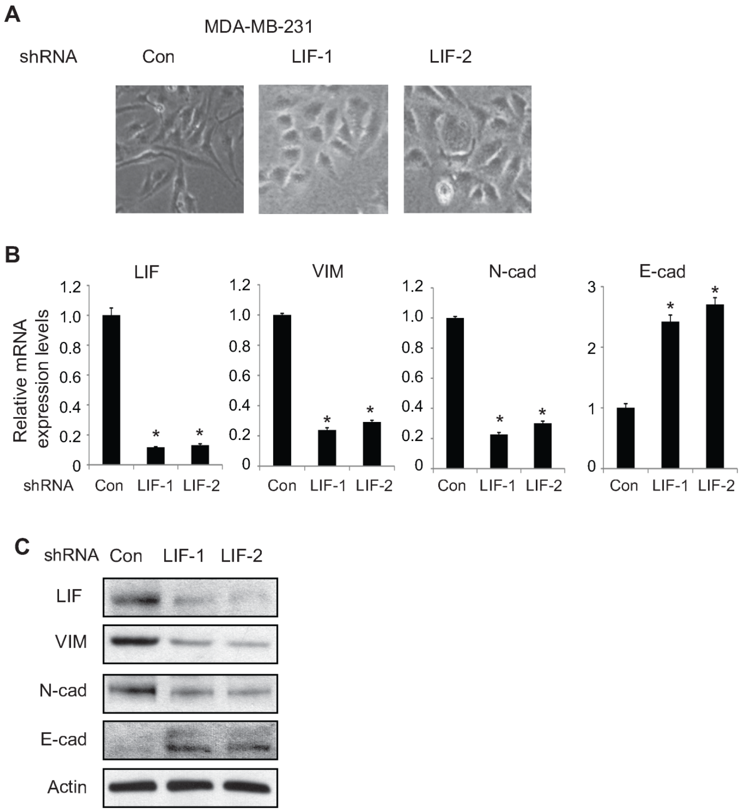

Supplementary Figure S4: Stable knockdown of endogenous LIF reverses EMT in MDA-MB-231 cells. (**A**) MDA-MB-231 cells with stable knockdown of LIF by shRNA vectors and their control cells were employed to examine EMT morphological change by phase-contrast photomicrographs. (**B and C**) Stable knockdown of LIF changed the mRNA (B) and protein (C) levels of EMT markers in MDA-MB-231 cells. In (B) data are presented as mean ± s.d. ($n = 3$). *$p < 0.05$; student t-test.

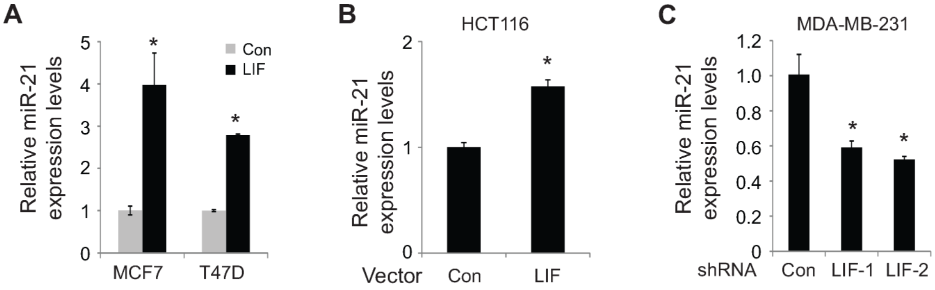

Supplementary Figure S5: LIF induces the expression of miR-21 in cells. (**A**) The expression of miR-21 was determined in MCF7 and T47D cells with ectopic stable LIF expression and their control cells. (**B**) The expression of miR-21 was determined in HCT116 cells with ectopic expression of LIF. (**C**) The expression of miR-21 was determined in MDA-MB-231 cells with stable knockdown of LIF by shRNA vectors targeting LIF and their control cells. The expression of miR-21 was normalized to the U6 snRNA. Data are presented as mean ± s.d. ($n = 3$). *$p < 0.05$, student t-test.

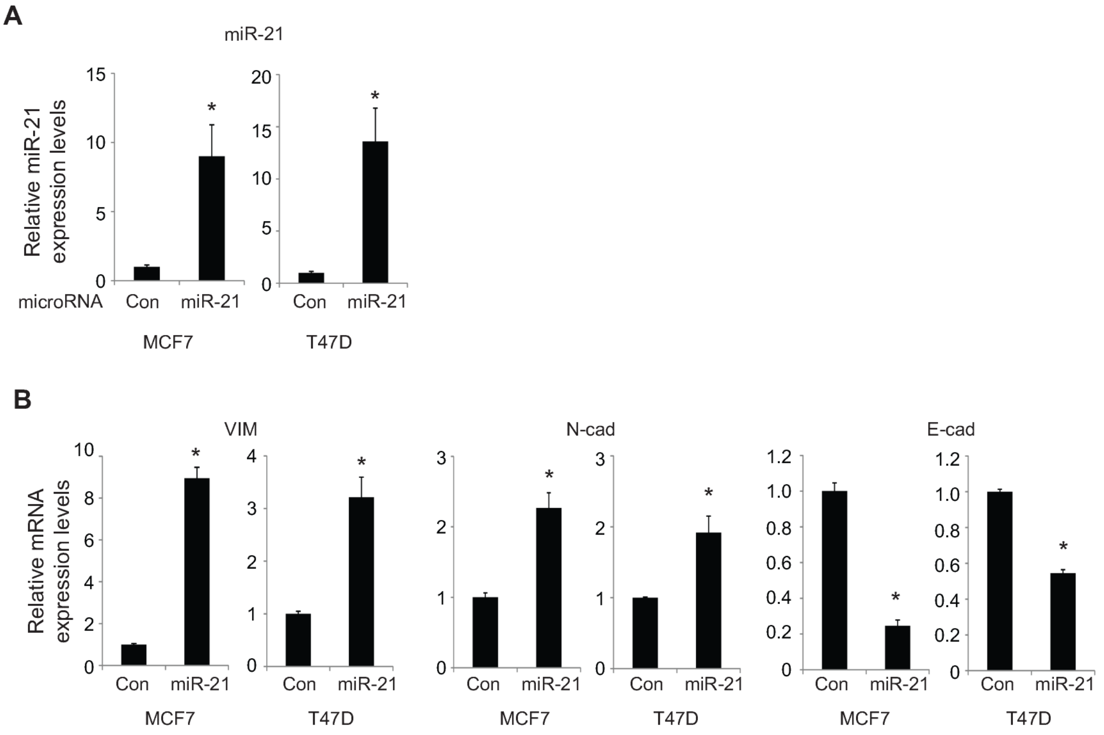

Supplementary Figure S6: miR-21 increases the mRNA expression levels of mesenchymal markers and decreased the expression levels of epithelial marker. MCF7 and T47D cells were transfected with miR-21 mimics or microRNA controls. The expression levels of mesenchymal markers (VIM and N-cad), and epithelial marker (E-cad) were determined by real-time PCR. The mRNA levels of these genes were normalized to β-actin. Data are presented as mean ± s.d. (n = 3). *$p < 0.05$; student t-test.

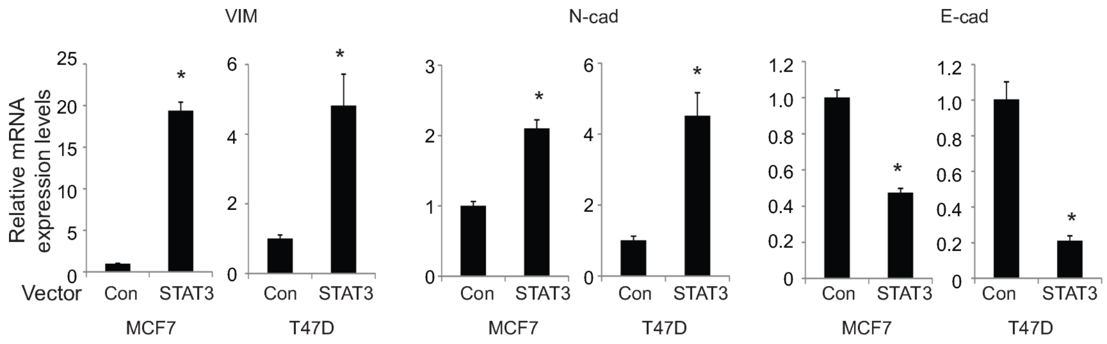

Supplementary Figure S7: STAT3 increases the mRNA expression levels of mesenchymal markers and decreased the expression levels of the epithelial marker. MCF7 and T47D cells were transfected with STAT3 expression vectors or control vectors. The expression levels of mesenchymal markers (VIM and N-cad) and epithelial marker (E-cad) were determined by realtime PCR. The mRNA levels of these genes were normalized to β-actin. Data are presented as mean ± s.d. (n = 3). *$p < 0.05$; student t-test.

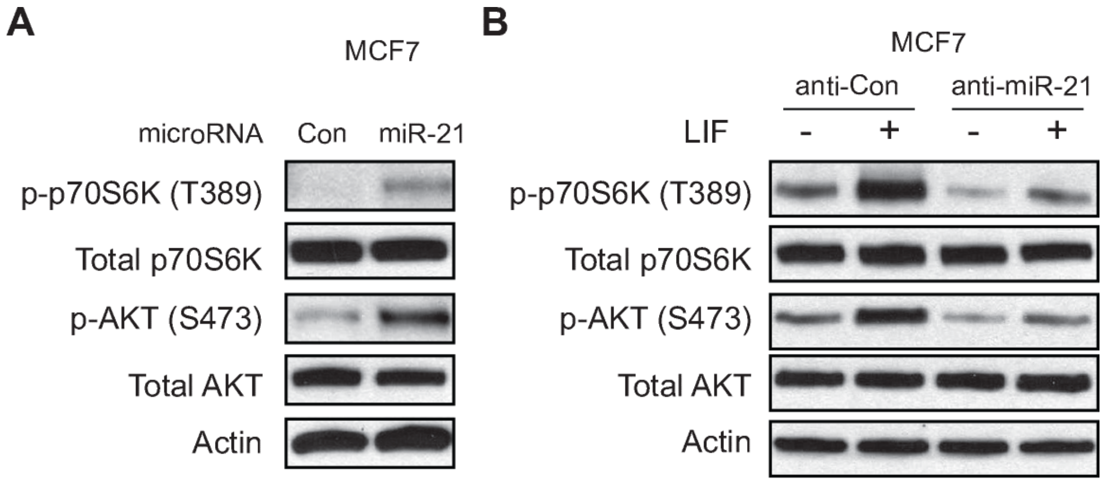

Supplementary Figure S8: Blocking miR-21 greatly inhibits the activation of the AKT/mTOR signaling by LIF. (**A**) miR-21 increased the phosphorylation levels of AKT and p70S6K in MCF7 cells. MCF7 cells were transfected with miR-21 mimics or microRNA controls, and the levels of phosphorylated AKT at S473 (p-AKT), total AKT, phosphorylated p70S6K at T389 (p-p70S6K) and total p70S6K were determined by Western blot assay. (**B**) Blocking miR-21 by anti-miR-21 oligos greatly inhibited the activation the p-AKT and p-p70S6K by LIF in MCF7 cells. MCF7 cells with and without stable ectopic expression of LIF were transfected with anti-miR-21 RNA or control RNA oligos.
